# Dr. Isaac R. Williams

**Published:** 1912-02

**Authors:** 


Dr. Isaac R. Williams, died January 21, 1912, in Rochester, quite suddenly. He was a soldier of the Civil War. Later, he practiced in Binghamton and subsequently in Walton.

